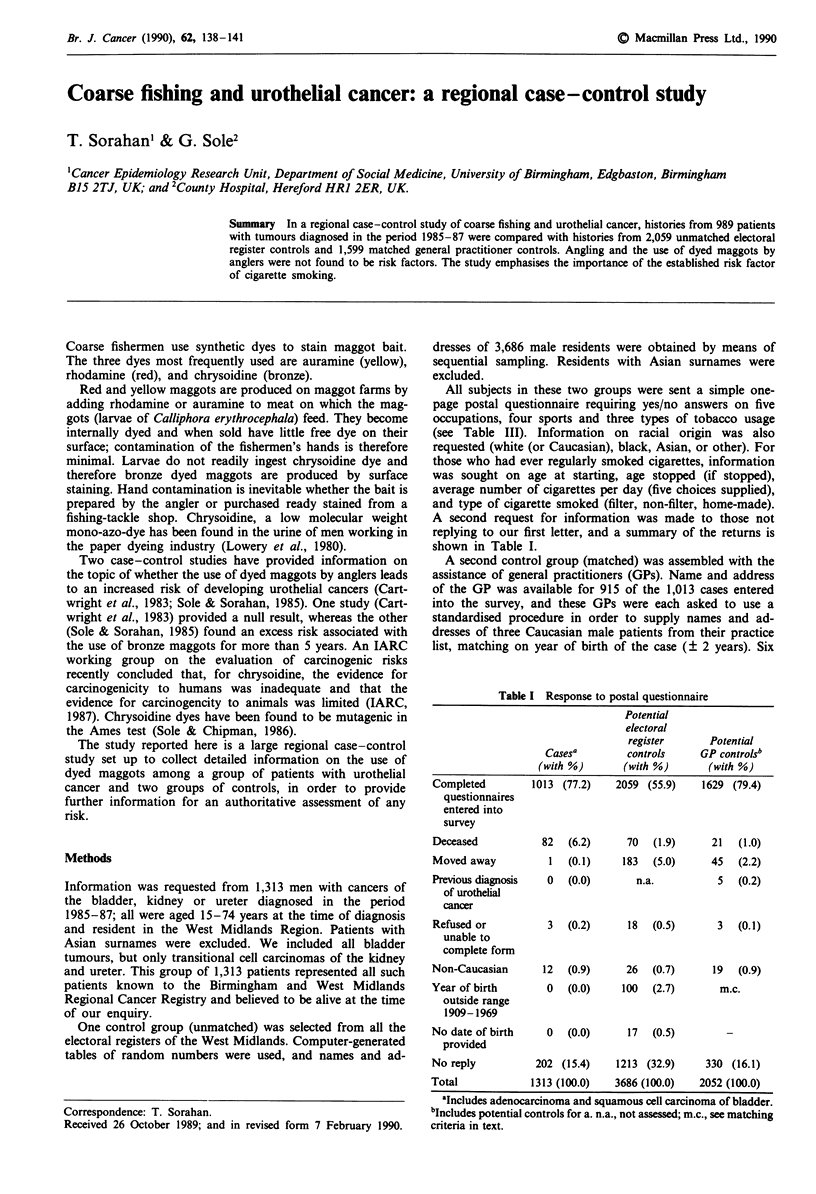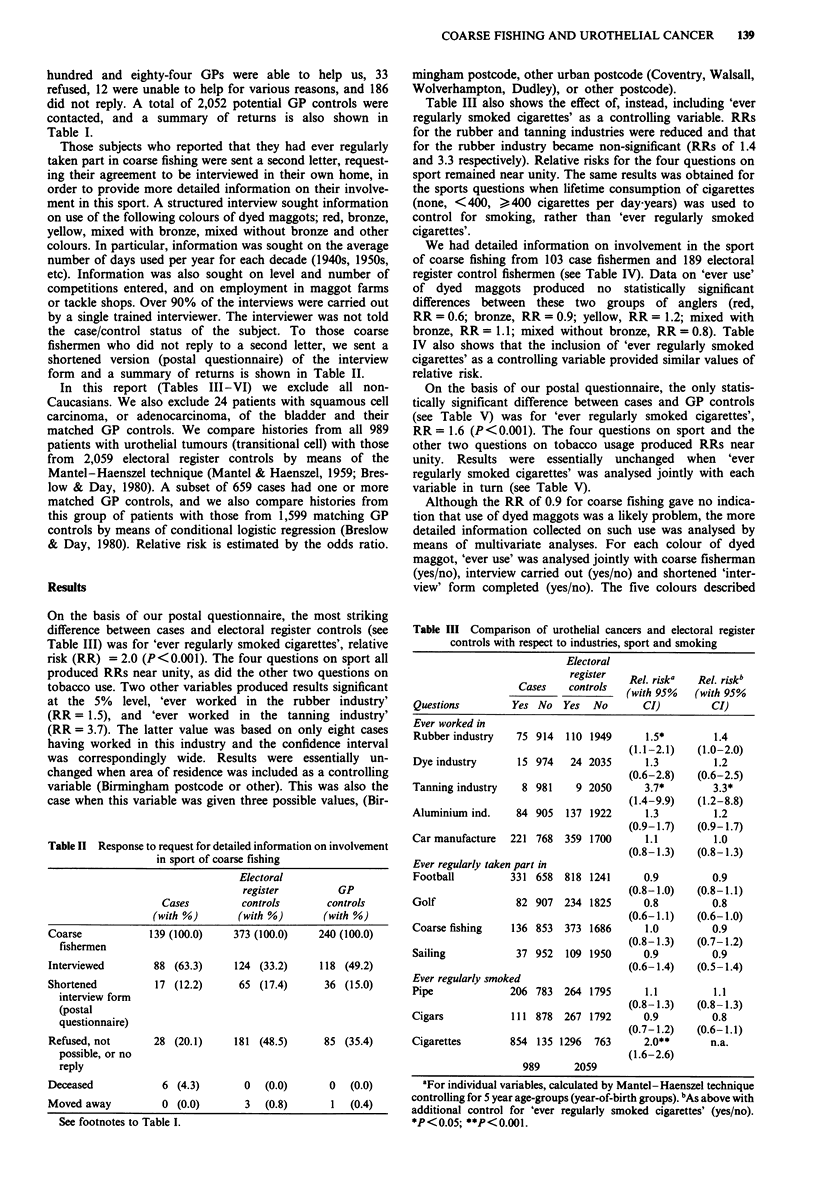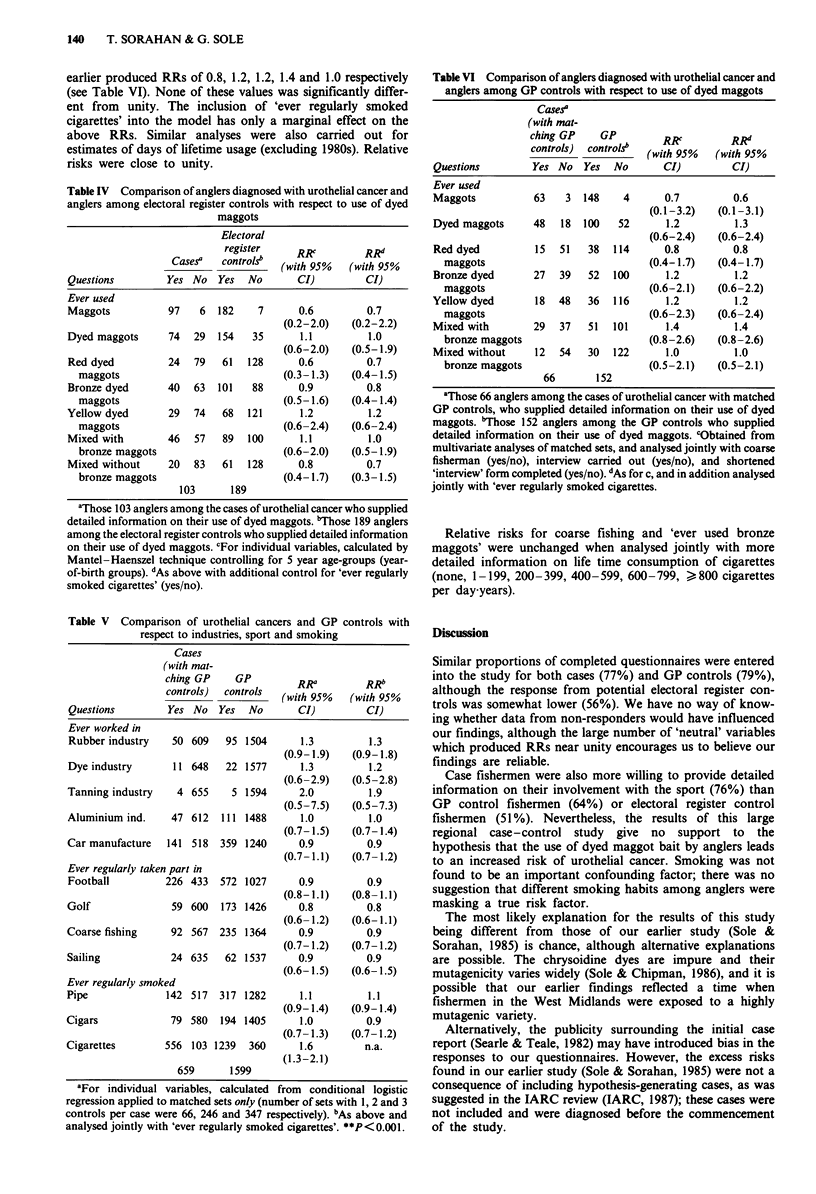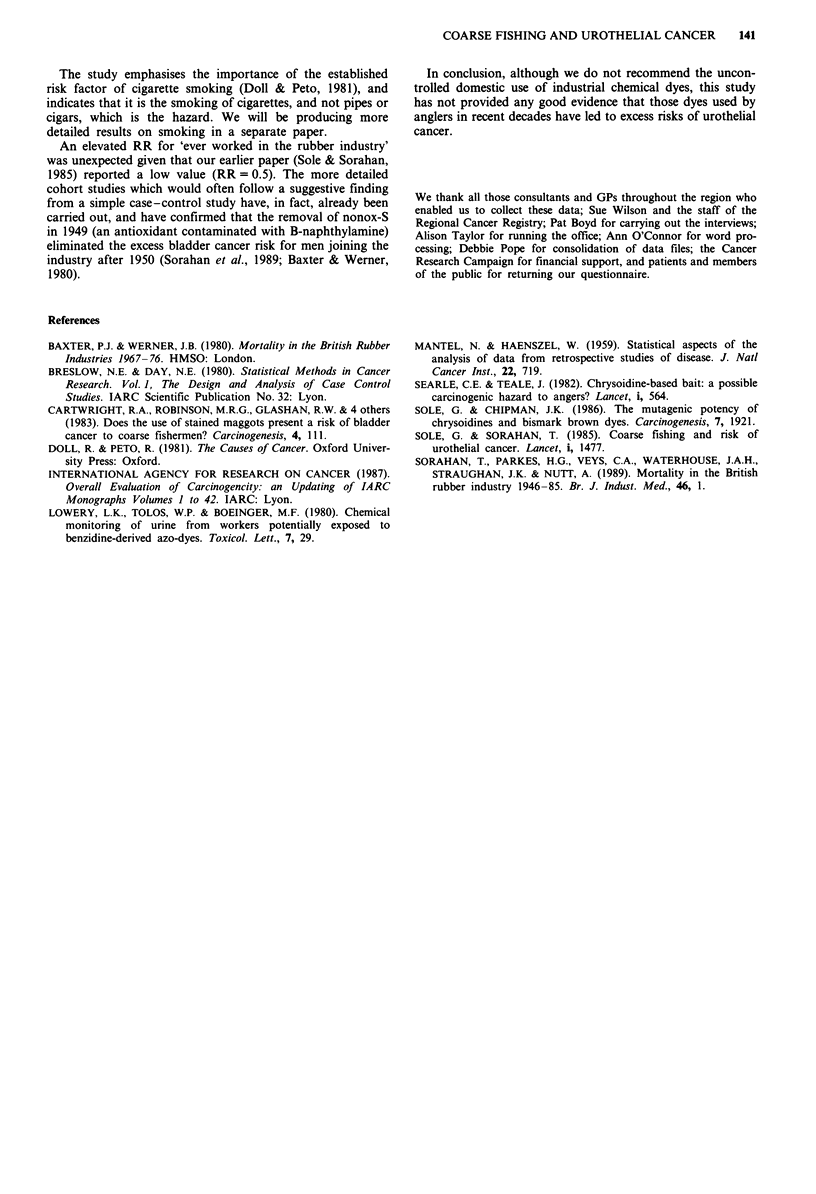# Coarse fishing and urothelial cancer: a regional case-control study.

**DOI:** 10.1038/bjc.1990.246

**Published:** 1990-07

**Authors:** T. Sorahan, G. Sole

**Affiliations:** Department of Social Medicine, University of Birmingham, Edgbaston, UK.

## Abstract

In a regional case-control study of coarse fishing and urothelial cancer, histories from 989 patients with tumours diagnosed in the period 1985-87 were compared with histories from 2,059 unmatched electoral register controls and 1,599 matched general practitioner controls. Angling and the use of dyed maggots by anglers were not found to be risk factors. The study emphasises the importance of the established risk factor of cigarette smoking.


Br. J. Cancer (1990), 62, 138-141 © Macmillan Press Ltd., 1990

### Coarse fishing and urothelial cancer: a regional case-control study

T. Sorahan[1] & G. Sole[2]

[1]*Cancer Epidemiology Research Unit, Department of Social Medicine, University of Birmingham, Edgbaston, Birmingham B15 2TJ, UK; and* [2]*County Hospital, Hereford HR1 2ER, UK.*

**Summary** In a regional case-control study of coarse fishing and urothelial cancer, histories from 989 patients with tumours diagnosed in the period 1985-87 were compared with histories from 2,059 unmatched electoral register controls and 1,599 matched general practitioner controls. Angling and the use of dyed maggots by anglers were not found to be risk factors. The study emphasises the importance of the established risk factor of cigarette smoking.

Coarse fishermen use synthetic dyes to stain maggot bait. The three dyes most frequently used are auramine (yellow), rhodamine (red), and chrysoidine (bronze).

Red and yellow maggots are produced on maggot farms by adding rhodamine or auramine to meat on which the maggots (larvae of *Calliphora erythrocephala*) feed. They become internally dyed and when sold have little free dye on their surface; contamination of the fishermen's hands is therefore minimal. Larvae do not readily ingest chrysoidine dye and therefore bronze dyed maggots are produced by surface staining. Hand contamination is inevitable whether the bait is prepared by the angler or purchased ready stained from a fishing-tackle shop. Chrysoidine, a low molecular weight mono-azo-dye has been found in the urine of men working in the paper dyeing industry (Lowery *et al.*, 1980).

Two case-control studies have provided information on the topic of whether the use of dyed maggots by anglers leads to an increased risk of developing urothelial cancers (Cartwright *et al.*, 1983; Sole & Sorahan, 1985). One study (Cartwright *et al.*, 1983) provided a null result, whereas the other (Sole & Sorahan, 1985) found an excess risk associated with the use of bronze maggots for more than 5 years. An IARC working group on the evaluation of carcinogenic risks recently concluded that, for chrysoidine, the evidence for carcinogenicity to humans was inadequate and that the evidence for carcinogencity to animals was limited (IARC, 1987). Chrysoidine dyes have been found to be mutagenic in the Ames test (Sole & Chipman, 1986).

The study reported here is a large regional case-control study set up to collect detailed information on the use of dyed maggots among a group of patients with urothelial cancer and two groups of controls, in order to provide further information for an authoritative assessment of any risk.

### Methods

Information was requested from 1,313 men with cancers of the bladder, kidney or ureter diagnosed in the period 1985-87; all were aged 15-74 years at the time of diagnosis and resident in the West Midlands Region. Patients with Asian surnames were excluded. We included all bladder tumours, but only transitional cell carcinomas of the kidney and ureter. This group of 1,313 patients represented all such patients known to the Birmingham and West Midlands Regional Cancer Registry and believed to be alive at the time of our enquiry.

One control group (unmatched) was selected from all the electoral registers of the West Midlands. Computer-generated tables of random numbers were used, and names and addresses of 3,686 male residents were obtained by means of sequential sampling. Residents with Asian surnames were excluded.

All subjects in these two groups were sent a simple one-page postal questionnaire requiring yes/no answers on five occupations, four sports and three types of tobacco usage (see Table III). Information on racial origin was also requested (white (or Caucasian), black, Asian, or other). For those who had ever regularly smoked cigarettes, information was sought on age at starting, age stopped (if stopped), average number of cigarettes per day (five choices supplied), and type of cigarette smoked (filter, non-filter, home-made). A second request for information was made to those not replying to our first letter, and a summary of the returns is shown in Table I.

A second control group (matched) was assembled with the assistance of general practitioners (GPs). Name and address of the GP was available for 915 of the 1,013 cases entered into the survey, and these GPs were each asked to use a standardised procedure in order to supply names and addresses of three Caucasian male patients from their practice list, matching on year of birth of the case (± 2 years). Six

**Table I** Response to postal questionnaire

| | *Cases*[a] *(with %)* | *Potential electoral register controls (with %)* | *Potential GP controls*[b] *(with %)* |
|---|---|---|---|
| Completed questionnaires entered into survey | 1013 (77.2) | 2059 (55.9) | 1629 (79.4) |
| Deceased | 82 (6.2) | 70 (1.9) | 21 (1.0) |
| Moved away | 1 (0.1) | 183 (5.0) | 45 (2.2) |
| Previous diagnosis of urothelial cancer | 0 (0.0) | n.a. | 5 (0.2) |
| Refused or unable to complete form | 3 (0.2) | 18 (0.5) | 3 (0.1) |
| Non-Caucasian | 12 (0.9) | 26 (0.7) | 19 (0.9) |
| Year of birth outside range 1909-1969 | 0 (0.0) | 100 (2.7) | m.c. |
| No date of birth provided | 0 (0.0) | 17 (0.5) | - |
| No reply | 202 (15.4) | 1213 (32.9) | 330 (16.1) |
| Total | 1313 (100.0) | 3686 (100.0) | 2052 (100.0) |

[a]Includes adenocarcinoma and squamous cell carcinoma of bladder. [b]Includes potential controls for a. n.a., not assessed; m.c., see matching criteria in text.

Correspondence: T. Sorahan.
Received 26 October 1989; and in revised form 7 February 1990.

hundred and eighty-four GPs were able to help us, 33 refused, 12 were unable to help for various reasons, and 186 did not reply. A total of 2,052 potential GP controls were contacted, and a summary of returns is also shown in Table I.

Those subjects who reported that they had ever regularly taken part in coarse fishing were sent a second letter, requesting their agreement to be interviewed in their own home, in order to provide more detailed information on their involvement in this sport. A structured interview sought information on use of the following colours of dyed maggots; red, bronze, yellow, mixed with bronze, mixed without bronze and other colours. In particular, information was sought on the average number of days used per year for each decade (1940s, 1950s, etc). Information was also sought on level and number of competitions entered, and on employment in maggot farms or tackle shops. Over 90% of the interviews were carried out by a single trained interviewer. The interviewer was not told the case/control status of the subject. To those coarse fishermen who did not reply to a second letter, we sent a shortened version (postal questionnaire) of the interview form and a summary of returns is shown in Table II.

In this report (Tables III–VI) we exclude all non-Caucasians. We also exclude 24 patients with squamous cell carcinoma, or adenocarcinoma, of the bladder and their matched GP controls. We compare histories from all 989 patients with urothelial tumours (transitional cell) with those from 2,059 electoral register controls by means of the Mantel–Haenszel technique (Mantel & Haenszel, 1959; Breslow & Day, 1980). A subset of 659 cases had one or more matched GP controls, and we also compare histories from this group of patients with those from 1,599 matching GP controls by means of conditional logistic regression (Breslow & Day, 1980). Relative risk is estimated by the odds ratio.

### Results

On the basis of our postal questionnaire, the most striking difference between cases and electoral register controls (see Table III) was for 'ever regularly smoked cigarettes', relative risk (RR) = 2.0 ($P<0.001$). The four questions on sport all produced RRs near unity, as did the other two questions on tobacco use. Two other variables produced results significant at the 5% level, 'ever worked in the rubber industry' (RR = 1.5), and 'ever worked in the tanning industry' (RR = 3.7). The latter value was based on only eight cases having worked in this industry and the confidence interval was correspondingly wide. Results were essentially unchanged when area of residence was included as a controlling variable (Birmingham postcode or other). This was also the case when this variable was given three possible values, (Birmingham postcode, other urban postcode (Coventry, Walsall, Wolverhampton, Dudley), or other postcode).

Table III also shows the effect of, instead, including 'ever regularly smoked cigarettes' as a controlling variable. RRs for the rubber and tanning industries were reduced and that for the rubber industry became non-significant (RRs of 1.4 and 3.3 respectively). Relative risks for the four questions on sport remained near unity. The same results was obtained for the sports questions when lifetime consumption of cigarettes (none, <400, ⩾400 cigarettes per day·years) was used to control for smoking, rather than 'ever regularly smoked cigarettes'.

We had detailed information on involvement in the sport of coarse fishing from 103 case fishermen and 189 electoral register control fishermen (see Table IV). Data on 'ever use' of dyed maggots produced no statistically significant differences between these two groups of anglers (red, RR = 0.6; bronze, RR = 0.9; yellow, RR = 1.2; mixed with bronze, RR = 1.1; mixed without bronze, RR = 0.8). Table IV also shows that the inclusion of 'ever regularly smoked cigarettes' as a controlling variable provided similar values of relative risk.

On the basis of our postal questionnaire, the only statistically significant difference between cases and GP controls (see Table V) was for 'ever regularly smoked cigarettes', RR = 1.6 ($P<0.001$). The four questions on sport and the other two questions on tobacco usage produced RRs near unity. Results were essentially unchanged when 'ever regularly smoked cigarettes' was analysed jointly with each variable in turn (see Table V).

Although the RR of 0.9 for coarse fishing gave no indication that use of dyed maggots was a likely problem, the more detailed information collected on such use was analysed by means of multivariate analyses. For each colour of dyed maggot, 'ever use' was analysed jointly with coarse fisherman (yes/no), interview carried out (yes/no) and shortened 'interview' form completed (yes/no). The five colours described

**Table II** Response to request for detailed information on involvement in sport of coarse fishing

| | *Cases (with %)* | *Electoral register controls (with %)* | *GP controls (with %)* |
|---|---|---|---|
| Coarse fishermen | 139 (100.0) | 373 (100.0) | 240 (100.0) |
| Interviewed | 88 (63.3) | 124 (33.2) | 118 (49.2) |
| Shortened interview form (postal questionnaire) | 17 (12.2) | 65 (17.4) | 36 (15.0) |
| Refused, not possible, or no reply | 28 (20.1) | 181 (48.5) | 85 (35.4) |
| Deceased | 6 (4.3) | 0 (0.0) | 0 (0.0) |
| Moved away | 0 (0.0) | 3 (0.8) | 1 (0.4) |

See footnotes to Table I.

**Table III** Comparison of urothelial cancers and electoral register controls with respect to industries, sport and smoking

| | *Cases* | | *Electoral register controls* | | | |
|---|---|---|---|---|---|---|
| *Questions* | *Yes* | *No* | *Yes* | *No* | *Rel. risk[a] (with 95% CI)* | *Rel. risk[b] (with 95% CI)* |
| *Ever worked in* | | | | | | |
| Rubber industry | 75 | 914 | 110 | 1949 | 1.5* (1.1–2.1) | 1.4 (1.0–2.0) |
| Dye industry | 15 | 974 | 24 | 2035 | 1.3 (0.6–2.8) | 1.2 (0.6–2.5) |
| Tanning industry | 8 | 981 | 9 | 2050 | 3.7* (1.4–9.9) | 3.3* (1.2–8.8) |
| Aluminium ind. | 84 | 905 | 137 | 1922 | 1.3 (0.9–1.7) | 1.2 (0.9–1.7) |
| Car manufacture | 221 | 768 | 359 | 1700 | 1.1 (0.8–1.3) | 1.0 (0.8–1.3) |
| *Ever regularly taken part in* | | | | | | |
| Football | 331 | 658 | 818 | 1241 | 0.9 (0.8–1.0) | 0.9 (0.8–1.1) |
| Golf | 82 | 907 | 234 | 1825 | 0.8 (0.6–1.1) | 0.8 (0.6–1.0) |
| Coarse fishing | 136 | 853 | 373 | 1686 | 1.0 (0.8–1.3) | 0.9 (0.7–1.2) |
| Sailing | 37 | 952 | 109 | 1950 | 0.9 (0.6–1.4) | 0.9 (0.5–1.4) |
| *Ever regularly smoked* | | | | | | |
| Pipe | 206 | 783 | 264 | 1795 | 1.1 (0.8–1.3) | 1.1 (0.8–1.3) |
| Cigars | 111 | 878 | 267 | 1792 | 0.9 (0.7–1.2) | 0.8 (0.6–1.1) |
| Cigarettes | 854 | 135 | 1296 | 763 | 2.0** (1.6–2.6) | n.a. |
| | 989 | | 2059 | | | |

[a]For individual variables, calculated by Mantel–Haenszel technique controlling for 5 year age-groups (year-of-birth groups). [b]As above with additional control for 'ever regularly smoked cigarettes' (yes/no). *$P<0.05$; **$P<0.001$.

earlier produced RRs of 0.8, 1.2, 1.2, 1.4 and 1.0 respectively (see Table VI). None of these values was significantly different from unity. The inclusion of 'ever regularly smoked cigarettes' into the model has only a marginal effect on the above RRs. Similar analyses were also carried out for estimates of days of lifetime usage (excluding 1980s). Relative risks were close to unity.

**Table IV** Comparison of anglers diagnosed with urothelial cancer and anglers among electoral register controls with respect to use of dyed maggots

| Questions | Cases[a] Yes | Cases[a] No | Electoral register controls[b] Yes | Electoral register controls[b] No | RR[c] (with 95% CI) | RR[d] (with 95% CI) |
|---|---|---|---|---|---|---|
| *Ever used* | | | | | | |
| Maggots | 97 | 6 | 182 | 7 | 0.6 (0.2–2.0) | 0.7 (0.2–2.2) |
| Dyed maggots | 74 | 29 | 154 | 35 | 1.1 (0.6–2.0) | 1.0 (0.5–1.9) |
| Red dyed maggots | 24 | 79 | 61 | 128 | 0.6 (0.3–1.3) | 0.7 (0.4–1.5) |
| Bronze dyed maggots | 40 | 63 | 101 | 88 | 0.9 (0.5–1.6) | 0.8 (0.4–1.4) |
| Yellow dyed maggots | 29 | 74 | 68 | 121 | 1.2 (0.6–2.4) | 1.2 (0.6–2.4) |
| Mixed with bronze maggots | 46 | 57 | 89 | 100 | 1.1 (0.6–2.0) | 1.0 (0.5–1.9) |
| Mixed without bronze maggots | 20 | 83 | 61 | 128 | 0.8 (0.4–1.7) | 0.7 (0.3–1.5) |
| | 103 | | 189 | | | |

[a]Those 103 anglers among the cases of urothelial cancer who supplied detailed information on their use of dyed maggots. [b]Those 189 anglers among the electoral register controls who supplied detailed information on their use of dyed maggots. [c]For individual variables, calculated by Mantel–Haenszel technique controlling for 5 year age-groups (year-of-birth groups). [d]As above with additional control for 'ever regularly smoked cigarettes' (yes/no).

**Table V** Comparison of urothelial cancers and GP controls with respect to industries, sport and smoking

| Questions | Cases (with matching GP controls) Yes | Cases (with matching GP controls) No | GP controls Yes | GP controls No | RR[a] (with 95% CI) | RR[b] (with 95% CI) |
|---|---|---|---|---|---|---|
| *Ever worked in* | | | | | | |
| Rubber industry | 50 | 609 | 95 | 1504 | 1.3 (0.9–1.9) | 1.3 (0.9–1.8) |
| Dye industry | 11 | 648 | 22 | 1577 | 1.3 (0.6–2.9) | 1.2 (0.5–2.8) |
| Tanning industry | 4 | 655 | 5 | 1594 | 2.0 (0.5–7.5) | 1.9 (0.5–7.3) |
| Aluminium ind. | 47 | 612 | 111 | 1488 | 1.0 (0.7–1.5) | 1.0 (0.7–1.4) |
| Car manufacture | 141 | 518 | 359 | 1240 | 0.9 (0.7–1.1) | 0.9 (0.7–1.2) |
| *Ever regularly taken part in* | | | | | | |
| Football | 226 | 433 | 572 | 1027 | 0.9 (0.8–1.1) | 0.9 (0.8–1.1) |
| Golf | 59 | 600 | 173 | 1426 | 0.8 (0.6–1.2) | 0.8 (0.6–1.1) |
| Coarse fishing | 92 | 567 | 235 | 1364 | 0.9 (0.7–1.2) | 0.9 (0.7–1.2) |
| Sailing | 24 | 635 | 62 | 1537 | 0.9 (0.6–1.5) | 0.9 (0.6–1.5) |
| *Ever regularly smoked* | | | | | | |
| Pipe | 142 | 517 | 317 | 1282 | 1.1 (0.9–1.4) | 1.1 (0.9–1.4) |
| Cigars | 79 | 580 | 194 | 1405 | 1.0 (0.7–1.3) | 0.9 (0.7–1.2) |
| Cigarettes | 556 | 103 | 1239 | 360 | 1.6 (1.3–2.1) | n.a. |
| | 659 | | 1599 | | | |

[a]For individual variables, calculated from conditional logistic regression applied to matched sets *only* (number of sets with 1, 2 and 3 controls per case were 66, 246 and 347 respectively). [b]As above and analysed jointly with 'ever regularly smoked cigarettes'. $^{**}P<0.001$.

**Table VI** Comparison of anglers diagnosed with urothelial cancer and anglers among GP controls with respect to use of dyed maggots

| Questions | Cases[a] (with matching GP controls) Yes | Cases[a] (with matching GP controls) No | GP controls[b] Yes | GP controls[b] No | RR[c] (with 95% CI) | RR[d] (with 95% CI) |
|---|---|---|---|---|---|---|
| *Ever used* | | | | | | |
| Maggots | 63 | 3 | 148 | 4 | 0.7 (0.1–3.2) | 0.6 (0.1–3.1) |
| Dyed maggots | 48 | 18 | 100 | 52 | 1.2 (0.6–2.4) | 1.3 (0.6–2.4) |
| Red dyed maggots | 15 | 51 | 38 | 114 | 0.8 (0.4–1.7) | 0.8 (0.4–1.7) |
| Bronze dyed maggots | 27 | 39 | 52 | 100 | 1.2 (0.6–2.1) | 1.2 (0.6–2.2) |
| Yellow dyed maggots | 18 | 48 | 36 | 116 | 1.2 (0.6–2.3) | 1.2 (0.6–2.4) |
| Mixed with bronze maggots | 29 | 37 | 51 | 101 | 1.4 (0.8–2.6) | 1.4 (0.8–2.6) |
| Mixed without bronze maggots | 12 | 54 | 30 | 122 | 1.0 (0.5–2.1) | 1.0 (0.5–2.1) |
| | 66 | | 152 | | | |

[a]Those 66 anglers among the cases of urothelial cancer with matched GP controls, who supplied detailed information on their use of dyed maggots. [b]Those 152 anglers among the GP controls who supplied detailed information on their use of dyed maggots. [c]Obtained from multivariate analyses of matched sets, and analysed jointly with coarse fisherman (yes/no), interview carried out (yes/no), and shortened 'interview' form completed (yes/no). [d]As for c, and in addition analysed jointly with 'ever regularly smoked cigarettes.

Relative risks for coarse fishing and 'ever used bronze maggots' were unchanged when analysed jointly with more detailed information on life time consumption of cigarettes (none, 1–199, 200–399, 400–599, 600–799, ⩾800 cigarettes per day·years).

### Discussion

Similar proportions of completed questionnaires were entered into the study for both cases (77%) and GP controls (79%), although the response from potential electoral register controls was somewhat lower (56%). We have no way of knowing whether data from non-responders would have influenced our findings, although the large number of 'neutral' variables which produced RRs near unity encourages us to believe our findings are reliable.

Case fishermen were also more willing to provide detailed information on their involvement with the sport (76%) than GP control fishermen (64%) or electoral register control fishermen (51%). Nevertheless, the results of this large regional case–control study give no support to the hypothesis that the use of dyed maggot bait by anglers leads to an increased risk of urothelial cancer. Smoking was not found to be an important confounding factor; there was no suggestion that different smoking habits among anglers were masking a true risk factor.

The most likely explanation for the results of this study being different from those of our earlier study (Sole & Sorahan, 1985) is chance, although alternative explanations are possible. The chrysoidine dyes are impure and their mutagenicity varies widely (Sole & Chipman, 1986), and it is possible that our earlier findings reflected a time when fishermen in the West Midlands were exposed to a highly mutagenic variety.

Alternatively, the publicity surrounding the initial case report (Searle & Teale, 1982) may have introduced bias in the responses to our questionnaires. However, the excess risks found in our earlier study (Sole & Sorahan, 1985) were not a consequence of including hypothesis-generating cases, as was suggested in the IARC review (IARC, 1987); these cases were not included and were diagnosed before the commencement of the study.

The study emphasises the importance of the established risk factor of cigarette smoking (Doll & Peto, 1981), and indicates that it is the smoking of cigarettes, and not pipes or cigars, which is the hazard. We will be producing more detailed results on smoking in a separate paper.

An elevated RR for 'ever worked in the rubber industry' was unexpected given that our earlier paper (Sole & Sorahan, 1985) reported a low value (RR = 0.5). The more detailed cohort studies which would often follow a suggestive finding from a simple case–control study have, in fact, already been carried out, and have confirmed that the removal of nonox-S in 1949 (an antioxidant contaminated with B-naphthylamine) eliminated the excess bladder cancer risk for men joining the industry after 1950 (Sorahan *et al.*, 1989; Baxter & Werner, 1980).

In conclusion, although we do not recommend the uncontrolled domestic use of industrial chemical dyes, this study has not provided any good evidence that those dyes used by anglers in recent decades have led to excess risks of urothelial cancer.

We thank all those consultants and GPs throughout the region who enabled us to collect these data; Sue Wilson and the staff of the Regional Cancer Registry; Pat Boyd for carrying out the interviews; Alison Taylor for running the office; Ann O'Connor for word processing; Debbie Pope for consolidation of data files; the Cancer Research Campaign for financial support, and patients and members of the public for returning our questionnaire.

### References

BAXTER, P.J. & WERNER, J.B. (1980). *Mortality in the British Rubber Industries 1967–76*. HMSO: London.

BRESLOW, N.E. & DAY, N.E. (1980). *Statistical Methods in Cancer Research. Vol. 1, The Design and Analysis of Case Control Studies*. IARC Scientific Publication No. 32: Lyon.

CARTWRIGHT, R.A., ROBINSON, M.R.G., GLASHAN, R.W. & 4 others (1983). Does the use of stained maggots present a risk of bladder cancer to coarse fishermen? *Carcinogenesis*, **4**, 111.

DOLL, R. & PETO, R. (1981). *The Causes of Cancer*. Oxford University Press: Oxford.

INTERNATIONAL AGENCY FOR RESEARCH ON CANCER (1987). *Overall Evaluation of Carcinogencity: an Updating of IARC Monographs Volumes 1 to 42*. IARC: Lyon.

LOWERY, L.K., TOLOS, W.P. & BOEINGER, M.F. (1980). Chemical monitoring of urine from workers potentially exposed to benzidine-derived azo-dyes. *Toxicol. Lett.*, **7**, 29.

MANTEL, N. & HAENSZEL, W. (1959). Statistical aspects of the analysis of data from retrospective studies of disease. *J. Natl Cancer Inst.*, **22**, 719.

SEARLE, C.E. & TEALE, J. (1982). Chrysoidine-based bait: a possible carcinogenic hazard to angers? *Lancet*, **i**, 564.

SOLE, G. & CHIPMAN, J.K. (1986). The mutagenic potency of chrysoidines and bismark brown dyes. *Carcinogenesis*, **7**, 1921.

SOLE, G. & SORAHAN, T. (1985). Coarse fishing and risk of urothelial cancer. *Lancet*, **i**, 1477.

SORAHAN, T., PARKES, H.G., VEYS, C.A., WATERHOUSE, J.A.H., STRAUGHAN, J.K. & NUTT, A. (1989). Mortality in the British rubber industry 1946–85. *Br. J. Indust. Med.*, **46**, 1.